# Structure and Calcium Binding Properties of a Neuronal Calcium-Myristoyl Switch Protein, Visinin-Like Protein 3

**DOI:** 10.1371/journal.pone.0165921

**Published:** 2016-11-07

**Authors:** Congmin Li, Sunghyuk Lim, Karl H. Braunewell, James B. Ames

**Affiliations:** 1 Department of Chemistry, University of California Davis, Davis, CA, United States of America; 2 Department of Neurophysiology, Medical Faculty, Ruhr University Bochum, Bochum, Germany; University of Alabama at Birmingham, UNITED STATES

## Abstract

Visinin-like protein 3 (VILIP-3) belongs to a family of Ca^2+^-myristoyl switch proteins that regulate signal transduction in the brain and retina. Here we analyze Ca^2+^ binding, characterize Ca^2+^-induced conformational changes, and determine the NMR structure of myristoylated VILIP-3. Three Ca^2+^ bind cooperatively to VILIP-3 at EF2, EF3 and EF4 (K_D_ = 0.52 μM and Hill slope of 1.8). NMR assignments, mutagenesis and structural analysis indicate that the covalently attached myristoyl group is solvent exposed in Ca^2+^-bound VILIP-3, whereas Ca^2+^-free VILIP-3 contains a sequestered myristoyl group that interacts with protein residues (E26, Y64, V68), which are distinct from myristate contacts seen in other Ca^2+^-myristoyl switch proteins. The myristoyl group in VILIP-3 forms an unusual L-shaped structure that places the C_14_ methyl group inside a shallow protein groove, in contrast to the much deeper myristoyl binding pockets observed for recoverin, NCS-1 and GCAP1. Thus, the myristoylated VILIP-3 protein structure determined in this study is quite different from those of other known myristoyl switch proteins (recoverin, NCS-1, and GCAP1). We propose that myristoylation serves to fine tune the three-dimensional structures of neuronal calcium sensor proteins as a means of generating functional diversity.

## Introduction

VILIP-3 is a neuronal calcium sensor (NCS) protein that belongs to the calmodulin superfamily of calcium sensors [1–4]. VILIP-3 is expressed in Purkinje cells of the cerebellum [5,6] and rat hippocampus [7,8], where it may regulate synaptic plasticity relevant for learning and memory. VILIP-3 is 94% identical in sequence to the NCS protein, hippocalcin that regulates Ca^2+^-dependent K^+^ channels involved in triggering slow afterhyperpolarization (sAHP) current important for spike frequency adaptation [9,10]. The physiological target of VILIP-3 is currently not known, but its high sequence identity to hippocalcin (94% identity) suggests that VILIP-3 may also interact with Ca^2+^-gated sAHP channels in hippocampal neurons. VILIP-3 has also been suggested to affect MAP kinase signaling [6].

**Fig 1:**
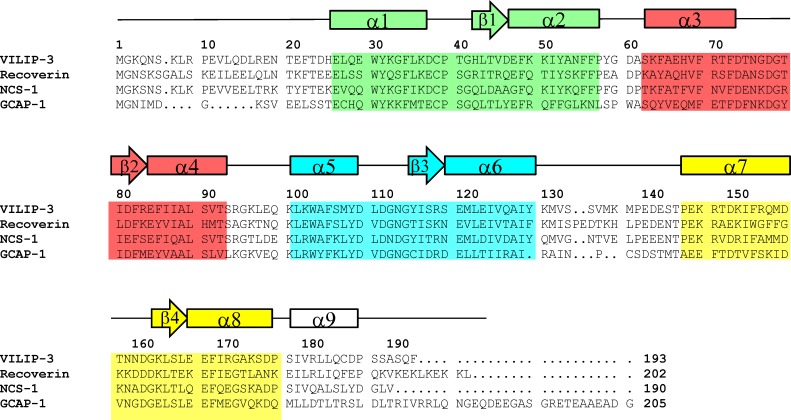
Amino acid sequence alignment of human VILIP-3 with other NCS proteins Secondary structure elements (helices and strands derived from NMR chemical shifts) and EF-hand motifs (EF1 green, EF2 red, EF3 cyan and EF4 yellow) are shown above the amino acid sequence of VILIP-3. Residues in VILIP-3 that interact with the myristoyl group are highlighted in bold and blue. Swiss Protein Database accession numbers are P37235 (human VILIP-3), P21457 (bovine recoverin), P62166 (human NCS-1), P46065 (bovine GCAP1).

VILIP-3 is structurally related to a family of Ca^2+^-myristoyl switch proteins that contain four EF-hand motifs and a covalently attached N-terminal myristoyl group (Fig 1). NMR and/or crystal structures are known for myristoylated forms of GCAP1 [11,12], NCS-1 [13], and recoverin [14,15]. The binding of Ca^2+^ to NCS proteins promotes their binding to cellular membranes [16–21]. The myristoyl group attached to recoverin is sequestered structurally inside the Ca^2+^-free protein [15], whereas the myristoyl group becomes solvent exposed in Ca^2+^ recoverin [14,21]. This Ca^2+^-dependent extrusion of the myristoyl group, referred to as a Ca^2+^-myristoyl switch, enables recoverin and VILIP-3 to bind cellular membrane targets only at high Ca^2+^ levels [22].

Here, we analyze the Ca^2+^ binding and folding energetics of VILIP-3, and present the NMR structure of myristoylated VILIP-3. VILIP-3 binds to 3 Ca^2+^ at saturation with an apparent dissociation constant (K_d_) of 0.52 μM and positive cooperativity (Hill slope = 1.8). NMR evidence demonstrates that the covalently attached myristoyl group is solvent exposed in Ca^2+^-bound VILIP-3. By contrast, the Ca^2+^-free VILIP-3 structure contains a long N-terminal loop that positions the myristoyl group inside a protein cavity that is structurally quite different from myristate binding sites seen in recoverin [15], NCS-1 [13] and GCAP1 [11,12]. The unusual structure of myristoylated VILIP-3 suggests that N-terminal myristoylation may serve to help mold each NCS protein into a unique fold [23].

## Materials and Methods

### Preparation and Purification of Recombinant VILIP-3

Human VILIP-3 gene was subcloned in pET3d(+). Site specific VILIP-3 mutants (Y64A and V68A) were generated by QuikChange site-directed mutagenesis kit (Stratagene). Bacterial cells for expressing recombinant myristoylated VILIP-3 protein were generated by co-transforming BL21(DE3) cells with both pET3d-VILIP and pBB131 vector encoding yeast N-myristoyltransferase.

The expression and purification of recombinant VILIP-3 has been described previously [24]. Expression of recombinant VILIP-3 protein and yeast N-myristoyltransferase were both induced by adding isopropyl β-D-1-thiogalactopyranoside (IPTG) to the cell culture at a final concentration of 0.5 mM (when the cell density reached OD_600_ = 0.5) and the cells were then grown at 25°C for 12–16 hr. Myristic acid (10 mg/L) was added exogeneously 1 hr before induction. Bacterial cells harvested by centrifugation from a 1-L culture typically contained ~10 mg of expressed myristoylated VILIP-3. The isolation and purification of myristoylated VILIP-3 was described previously [24]. The final purified myristoylated VILIP-3 protein was more than 95% pure as determined by SDS-PAGE. Final purified myristoylated VILIP-3 samples contained less than 5% of unmyristoylated protein as judged by reverse-phase HPLC.

### Isothermal Titration Calorimetry

A VP-ITC calorimeter (Micro-Cal) was used to measure Ca^2+^ binding data as described previously [25]. VILIP-3 protein (50 *μ*M) used for ITC studies was dissolved in 20 mM Tris buffer (pH 7.5), 50mM NaCl, 1 mM Tris (2-carboxyethyl) phosphine hydrochloride (TCEP). The precise protein concentration was determined by measuring optical density at 280-nm as described previously [24]. For each ITC titration, a total of 50 injections (5-*μ*L each) of 2.0 mM CaCl_2_ were added to the protein sample during the titration. All titrations were performed at 30°C.

### Differential Scanning Calorimetry

A VP-DSC calorimeter from MicroCal was used for all DSC measurements as described previously [25]. Each DSC scan used a temperature range of 10–110°C at a scan rate of 60°C/h. A buffer baseline was subtracted from each scan. Protein samples for DSC experiments consisted of either myristoylated and unmyristoylated VILIP-3 proteins (50 *μ*M) dissolved in 20 mM Tris buffer (pH 7.5) containing 100 mM NaCl and 1 mM β-mercaptoethanol with 2 mM CaCl_2_ (Ca^2+^-bound state) or 2 mM EDTA (Ca^2+^-free state).

### NMR Spectroscopy

NMR experiments were performed using Bruker Avance III 600 or 800 MHz spectrometers equipped with a triple-resonance TCI-cryoprobe probe. VILIP-3 samples for NMR were dissolved in 0.3 ml of 90% H_2_O, 10% [^2^H]H_2_O containing 10mM [^2^H_11_]Tris, pH 7.4, and 2 mM EDTA (apo-) or 5mM CaCl_2_ (Ca^2+^-bound). Two-dimensional ^1^H-^15^N HSQC spectra of VILIP-3 were recorded at 30°C as described previously [24]. Two dimensional ^1^H-^13^C HMQC and ^13^C(F1)-edited, ^13^C(F3)-filtered NOESY-HMQC experiments were recorded on VILIP-3 samples that contained a 99% ^13^C labeled myristoyl group as described previously [24]. All triple-resonance and ^13^C,^15^N-edited NOESY experiments were performed and analyzed as described by Clore et al. [26] on a sample of Ca^2+^-free ^13^C/^15^N-labeled myristoylated VILIP-3 (in 95% H_2_O, 5% ^2^H_2_O). All NMR data sets were processed and analyzed using NMRPipe [27] and Sparky. Sequence specific NMR assignments were described by [26].

### Structure Calculation

Multi-dimensional ^15^N-edited NOESY-HSQC and ^13^C-edited NOESY-HSQC spectra of Ca^2+^-free myristoylated VILIP-3 were acquired and analyzed as described previously [13]. A total of 1738 NOE distance restraints were derived from NOESY spectra. In addition to the NOE-derived distances, 156 distance constraints for 78 hydrogen bonds and 189 dihedral angle constraints (ϕ and ψ) were calculated using TALOS+ [28] and were used as restraints in the structure calculation. Fifty independent structures were calculated by XPLOR-NIH software [29] using the YASAP protocol [30,31] as described previously [32]. The 15 lowest energy structures were selected and overlaid with RMSD of 0.9 Å.

## Results

### Three Ca^2+^ Bind Cooperatively to VILIP-3

**Fig 2:**
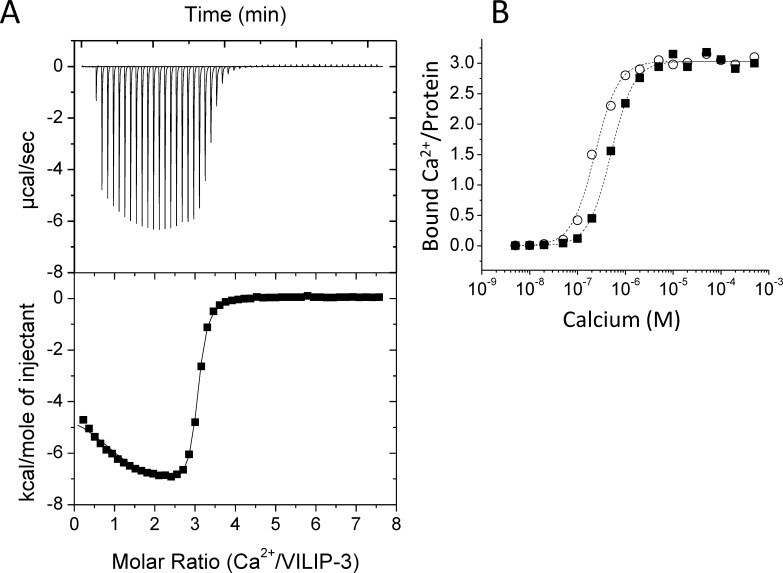
Ca^2+^ binding to VILIP-3. (A) Ca^2+^ binding ITC isotherm for myristoylated VILIP-3. The overall Ca^2+^ binding stoichiometry is 3:1, consistent with Ca^2+^ bound at EF2, EF3 and EF4. The average dissociation constant (K_d_) and binding enthalpy (ΔH) for the three sites are 0.3 μM and –6.4 kcal/mol, respectively. (B) Ca^2+^-binding data measured by flow dialysis [33]. Representative Ca^2+^ binding data for VILIP-3 wildtype (black squares) and Y64A (open circles) are shown. Fitted curves (dashed lines) were calculated using the Hill model. Fitting parameters for wild type and mutants are listed in Table 1.

Calcium binding to myristoylated VILIP-3 and mutants (E26A, F64A and V68A) were monitored by ITC (Fig 2A) and flow dialysis (Fig 2B). Optimal Ca^2+^ binding parameters are listed in Table 1. The ITC Ca^2+^-binding isotherm for wild type VILIP-3 exhibited exothermic binding of three Ca^2+^ with a steep Ca^2+^ dependence (K_D_ = 0.3 μM, ΔH = -6.9 kcal/mol). The fractional saturation (Y = [bound Ca^2+^]/[Protein]) was measured as a function of free Ca^2+^ concentration using flow-dialysis Ca^2+^ binding experiments (Fig 2B) as described by [33]. The fractional saturation (Y) was fit by the Hill equation:

$$Y=\frac{{\left[Ca^{2+}\right]}^{\alpha }}{{\left[Ca^{2+}\right]}^{\alpha }+K_{d}^{\alpha }}$$

Wild type VILIP-3 binds to Ca^2+^ with Hill coefficient (α) of 1.8 and apparent dissociation constant (K_D_) equal to 0.52 μM. The VILIP-3 mutants (E26A and F64A) each bound to Ca^2+^ with higher apparent affinity compared to wild type (Table 1), consistent with each mutant forming weaker myristate contacts in Ca^2+^-free VILIP-3. These mutants increase the Ca^2+^-binding affinity by destabilizing the Ca^2+^-free VILIP-3 structure (with sequestered myristoyl group) more so than the Ca^2+^-bound state (extruded myristate), which makes the free energy of Ca^2+^ binding more negative and hence more favorable. By contrast, the corresponding mutants in recoverin (E27A and Y65A) did not affect Ca^2+^ binding affinity (Table 1), which is consistent with both E26 and Y65 not making any contact with the myristate in the Ca^2+^-free and Ca^2+^-bound recoverin structures [14,15]. In summary, E26 and F64 of VILIP-3 make important contacts with the myristate (see below) and these contacts are not seen in recoverin.

**Table 1:** Ca^2+^ Binding and Folding Stability of VILIP-3, Recoverin and Mutants.

	Hill Coefficent (α)	K_D_ (μM)	T_M_ (°C)
VILIP-3^WT^	1.8 ±0.2	0.52 ±0.05	57 ±1
VILIP-3^WT,unmyr^	ND	ND	53 ±1
VILIP-3^E26A^	1.8 ±0.2	0.34 ±0.05	54 ±1
VILIP-3^E26A,unmyr^	ND	ND	54 ±1
VILIP-3^F64A^	1.8 ±0.2	0.23 ±0.05	53 ±1
VILIP-3^F64A,unmyr^	ND	ND	53 ±1
Recoverin^WT^	1.5 ±0.2	17 ±2	65 ±1
Recoverin^E27A^	1.5 ±0.2	20 ±2	64 ±1
Recoverin^Y65A^	1.5 ±0.2	19 ±2	63 ±1

Ca^2+^-binding data for myristoylated VILIP-3 were fit to the Hill model with Hill coefficient (α) and apparent dissociation constant (K_D_) in units of micromolar at 30°C. Ca^2+^-binding parameters were not determined (ND) for unmyristoylated VILIP-3. The unfolding temperatures (T_M_) of Ca^2+^-free forms of myristoylated VILIP-3, unmyristoylated VILIP-3 (unmyr), and myristoylated recoverin were determined by DSC in units of °C. VILIP-3 and Recoverin mutants are designated in the superscript.

### Myristoylation Increases Folding Stability of VILIP-3

**Fig 3:**
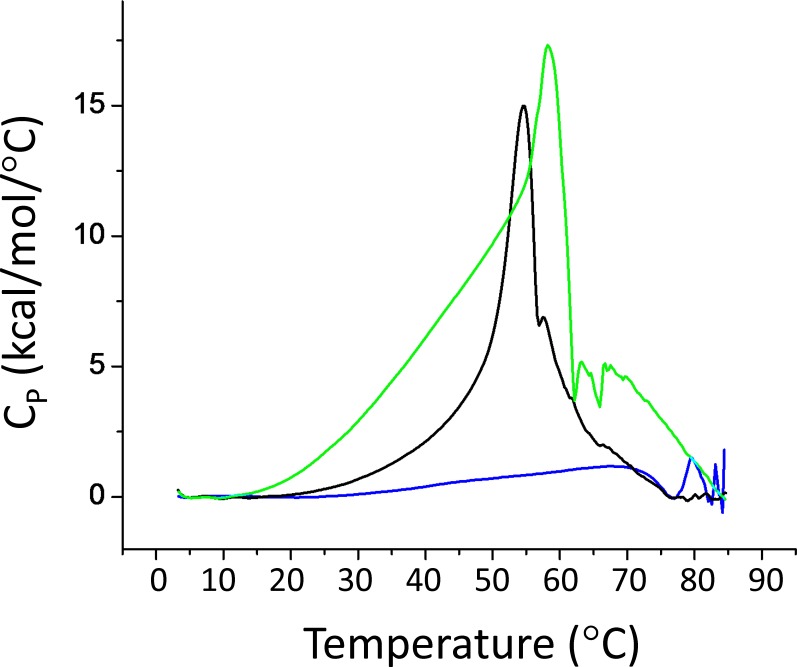
Folding stability of VILIP-3. Representative DSC thermograms for Ca^2+^-free VILIP-3 in both unmyristoylated (black) and myristoylated (green) states. The protein unfolding temperatures (T_M_) determined from the thermograms are listed in Table 1. The T_M_ for Ca^2+^-saturated myristoylated VILIP-3 could not be accurately measured due to complications caused by Ca^2+^-induced protein aggregation (see blue trace).

Differential scanning calorimetry (DSC) experiments were performed on VILIP-3 to measure the effect of myristoylation on protein folding stability. Representative DSC scans of wild type VILIP-3 are shown in Fig 3. The unfolding temperature of unmyristoylated Ca^2+^-free VILIP-3 (transition temperature, *T*_m_ = 53°C) is lower than the unfolding temperature of myristoylated VILIP-3 (T_m_ = 57°C), consistent with a stabilization caused by sequestration of the covalently attached myristoyl group inside Ca^2+^-free VILIP-3. The myristoylated VILIP-3 mutants (E26A and F64A) exhibited a detectably lower folding stability (T_m_ = 54°C, Table 1) compared to wild type, whereas the unmyristoylated mutants had the same folding stability as unmyristoylated wild type. The lower folding stability of myristoylated E26A and F64A is consistent with the side-chains of E26 and F64 both making important contacts with the myristoyl group in VILIP-3 as seen in the structure below. By comparison, the corresponding mutants in myristoylated recoverin (E27A and Y65A) did not affect the melting temperature (Table 1), which is consistent with E27 and Y65 both not making contact with the myristate in the recoverin structure [14].

For Ca^2+^ saturated myristoylated VILIP-3, the protein started to aggregate at around 42°C and the precise unfolding temperature could not be accurately measured by DSC (blue trace in Fig 3). The Ca^2+^-induced aggregation of VILIP-3 was most likely caused by Ca^2+^-induced exposure of the myristoyl group like that observed for recoverin [34].

### NMR Structure of Ca^2+^-free VILIP-3

**Fig 4:**
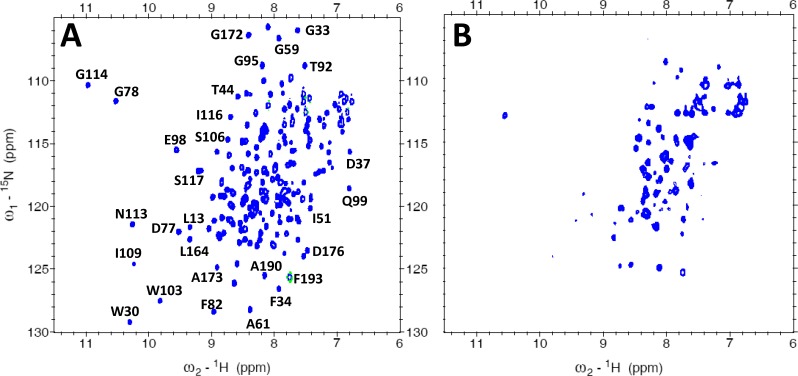
NMR spectroscopy of myristoylated VILIP-3. Two-dimensional (^1^H-^15^N HSQC) NMR spectra are shown for ^15^N-labeled VILIP-3 in the Ca^2+^-free (A) and Ca^2+^-bound (B) states. Spectra were recorded at 600 MHz at 30°C.

**Fig 5:**
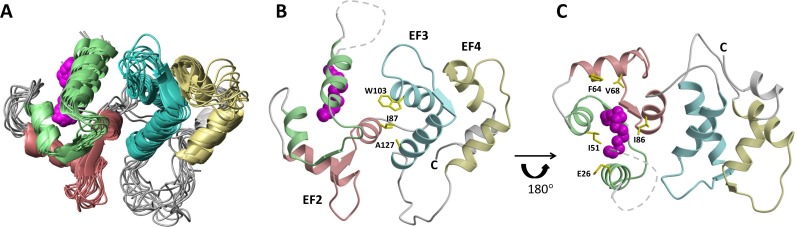
NMR-derived structures of myristoylated VILIP-3 (PDB ID: 5T7C). Ribbon diagrams show an overlay of the 10 lowest energy structures (A), and average main chain structure of Ca^2+^-free VILIP-3 (B) and rotated by 180° (C) with N-terminal myristoyl group highlighted magenta. The unstructured N-terminal loop region is depicted by a dashed line. Secondary structure elements (helices and strands) and EF-hand motifs are drawn in the same colors as in Fig 1 (EF1 green, EF2 red, EF3 cyan and EF4 yellow). Side-chain atoms of hydrophobic residues at the domain interface (A) and that contact the myristoyl group (B) are highlighted yellow.

^1^H-^15^N HSQC NMR spectra of Ca^2+^-free VILIP-3 (Fig 4A) exhibited a total of 223 highly dispersed peaks with uniform intensities, indicating that Ca^2+^-free VILIP-3 is monomeric under NMR conditions and stably folded. Sequence-specific NMR assignments of Ca^2+^-free myristoylated VILIP-3 were analyzed and described previously [35] (BMRB no. 18627). The assigned NMR resonances were used to analyze multi-dimensional NOESY spectra in order to obtain NOE distance restraints that defined the VILIP-3 structure. Secondary structure derived from the NMR data is illustrated in Fig 1. Three-dimensional protein structures of VILIP-3 were calculated based on NOE distance restraints and chemical shift analysis (see Methods). The final NMR-derived structures of Ca^2+^-free myristoylated VILIP-3 are illustrated in Fig 5 (PDB ID: 5T7C). Table 2 summarizes the structural statistics calculated for the 15 lowest-energy conformers.

**Table 2:** Structural Statistics for Ca^2+^-free VILIP-3.

**NMR restraints**	
Short range NOE (i to i + j, j = 1–4)	605
Long range NOE (i to i + j, j > 4)	271
Hydrogen bonds	124
Dihedral angle restraints	200
Protein-myristate	19
**RMSD to the mean coordinates (Å)**	
Backbone of structured regions^a^	0.90 ±0.09
Heavy atoms of structured regions	1.34 ±0.1
**RMSD from idealized geometry**	
Bond lengths (Å)	0.0064 ±0.0001
Bond angles (°)	2.00 ±0.0014
Impropers (°)	0.9 ±0.005
**Ramachandran statistics of 15 structures**	
Most favored regions (%)	82
Additional allowed regions (%)	15
Generously allowed regions (%)	3
Disallowed regions (%)	0

^a^Pairwise RMSD was calculated among 15 refined structures: residues in regions of regular secondary structure (24–35, 42–55, 61–72, 79–89, 97–108, 115–131, 146–156, 163–174, 178–184).

The entire polypeptide chain of Ca^2+^-free myristoylated VILIP-3 was defined by the NMR data, except for residues 4–20 and the last six residues at the C-terminus whose resonances were overlapped and/or exchange broadened. VILIP-3 contains a total of nine α-helices and four β-strands: α1 (residues 25–35), α2 (residues 46–56), α3 (residues 62–72), α4 (residues 82–91), α5 (residues 101–108), α6 (residues 118–129), α7 (residues 146–156), α8 (residues 166–176), α9 (residues 179–187), β1 (residues 42–44), β2 (residues 79–81), β3 (residues 115–117) and β4 (residues 163–165) (Fig 1A). The covalently attached myristoyl group at G2 is connected to a long unstructured loop (residues G2 –D24 depicted by the dashed line in Fig 5) that places the fatty acyl chain inside a shallow protein cavity formed by hydrophobic residues in EF1 and EF2 (residues L27, W30, F48, I51, Y52, Y64, V68, F82, F85, I86, L89). The myristoyl group interaction with protein residues (E26, F64, V68 and I86, highlighted yellow in Fig 5B) is unique to VILIP-3 and these contacts are not seen in structures of myristoylated recoverin [15], NCS-1 [13], or GCAP1 [11,12]. VILIP-3 contains two domains formed by the four EF-hands: EF1 (residues 26–55) and EF2 (residues 62–91) are connected together and form the N-domain; likewise, EF3 (residues 101–130) and EF4 (residues 146–175) form the C-domain. The domain interface is stabilized by residue contacts between EF2 (I87 and A88 in helix α4) and EF3 (W103, A104 and M107 in helix α5; and A127 and M131 in helix α6) that connect the two domains (see side-chains highlighted yellow in Fig 5A). Each EF-hand in VILIP-3 consists of a helix-turn-helix structure that is similar to the closed structure of Ca^2+^-free EF-hands seen previously in Ca^2+^-free recoverin [15] and apo-CaM [36]. The interhelical angles for the EF-hands of VILIP-3 are 142.8° (EF1), 123.7° (EF2), 120.1° (EF3) and 109.7° (EF4). The overall main chain structure of Ca^2+^-free myristoylated VILIP-3 (Fig 5) is quite different from the myristoylated forms of recoverin [15], GCAP1 [11], and NCS-1 [13]. A quantitative comparison of the main chain atoms of Ca^2+^-free VILIP-3 with those of recoverin, NCS-1 and GCAP1, indicates root-mean-squared deviations of 3.1, 3.6, and 3.9 Ǻ, respectively.

The HSQC NMR spectrum of Ca^2+^-bound myristoylated VILIP-3 (Fig 4B) looks quite different from that of Ca^2+^-free VILIP-3 (Fig 4A), consistent with a Ca^2+^-induced structural change. Fewer NMR peaks are detected for Ca^2+^-bound VILIP-3, perhaps due to spectral broadening caused by Ca^2+^-induced aggregation of the Ca^2+^-bound protein. The aggregation of Ca^2+^-bound myristoylated VILIP-3 is most likely caused by a Ca^2+^-induced extrusion of the covalently attached myristoyl group like that seen previously for recoverin [21,34].

### Myristoyl Binding Site in VILIP-3

**Fig 6:**
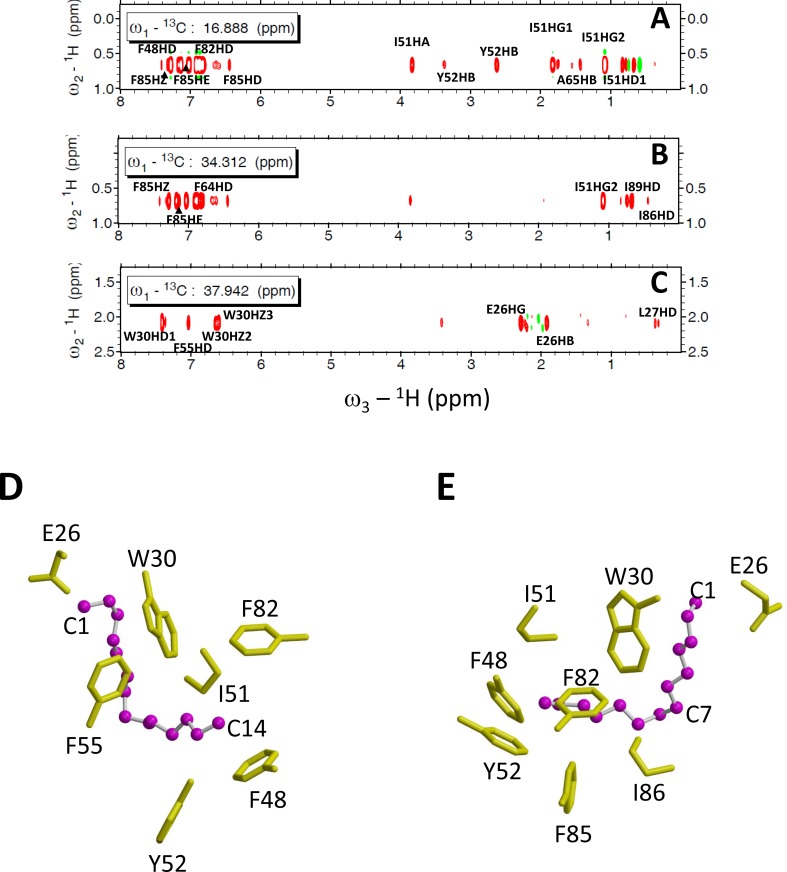
NMR spectroscopy of myristoyl binding site in VILIP-3. Three-dimensional ^13^C(F1)-edited ^13^C(F3)-filtered HMQC NOESY spectra of unlabeled VILIP-3 containing a ^13^C-labeled myristoyl group. (A-C) Two-dimensional slices of (^13^C/F1)-edited (^13^C/F3)-filtered HMQC NOESY spectra of ^13^C-labeled myristoyl group attached to Ca^2+^-free myristoylated VILIP-3 edited at ^13^C frequencies 16.89 (A), 34.31 (B) and 37.94 (C) ppm. NOE crosspeaks represent protons of myristate that are less than 5 Å away from aliphatic and aromatic resonances of the Ca^2+^-free protein (marked by residue labels). Schematic view of protein contacts along fatty acyl chain (D) and same view rotated by 180° (E). Side-chain atoms of hydrophobic residues near fatty acyl chain are highlighted yellow.

The structure of the covalently attached myristoyl group in VILIP-3 was probed by NMR experiments (3-D (^13^C/F_1_)-edited and (^13^C/F_3_)-filtered NOESY-HSQC) performed on Ca^2+^-free VILIP-3 samples that contained a ^13^C-labeled myristoyl group (Fig 6). These NMR spectra probed atoms in VILIP-3 located less than 5 Å away from the ^13^C-labeled fatty acyl chain. Representative Nuclear Overhauser effect (NOE) dipolar interactions are shown for the C_14_ methyl of the myristoyl group (^13^C_14_: F_2_ = 16.88 ppm, Fig 6A), C_12_ methylene (^13^C_12_: F_2_ = 34.31 ppm, Fig 6B), and the C_2_ methylene (^13^C_2_: F_2_ = 37.94 ppm, Fig 6C) of the myristoyl chain. The spectrum that probes the C_14_ methyl group (Fig 6A) reveals off-diagonal NMR resonances assigned to protein residues with aromatic ring protons (F48, F82 and F85) and aliphatic side-chains (I51, A65, V68 and I86). These NMR data imply that the C_14_ methyl group is surrounded by hydrophobic side-chains from residues in a protein pocket formed by the exiting helix of EF1 (F48, I51, Y52) and both helices of EF2 (F64, A65, V68, F82 and F85). The spectrum that probes the C_12_-position of the myristoyl moiety (Fig 6B) shows off-diagonal resonances assigned to protein residues in the exiting helix of EF2 (F85, I86 and I89). The spectrum that probes the C_2_-position of the myristoyl moiety (Fig 6C) shows off-diagonal resonances assigned to residues in EF1 (E26, L27 and W30). These NMR data reveal that the myristoyl group in VILIP-3 forms an unusual L-shaped structure with a 90° bend at C_7_ (Fig 6E) that positions the terminal C_14_-methyl group inside a protein cavity located in the N-terminal domain (see residues F48, I51, Y52, F82 and F85 in Fig 6D and 6E) that is quite different from the myristoyl group binding site in recoverin [15], GCAP1 [12] and NCS-1 [13]. The myristoyl group attached to VILIP-3 is about 40% buried inside the protein (Fig 6D and 6E). The C_14_ methyl group of the myristate makes close contacts with hydrophobic side-chains from F48, I51, Y52, F82, F85, I86 located inside the hydrophobic core (Fig 6D and 6E). The middle of the fatty acyl chain makes hydrophobic contacts with side-chains of Y52, F64, F85, I86 and I89. The carbonyl end of the myristate contacts the side-chains of E26 and W30 on the protein surface (Fig 6D). The environment around the myristoyl group in VILIP-3 consists of three amino acids (E26, F64, and V69) that do not make any myristate contacts in recoverin, GCAP1 or NCS-1, demonstrating that the myristate is located in a unique protein environment in VILIP-3. Indeed, alanine mutations of these myristate binding site amino acids in VILIP-3 (E26A, F64A and V69A) each affect Ca^2+^-binding affinity and folding stability of VILIP-3 (Table 1). The corresponding mutations in recoverin (E27A, Y65A and V69A) do not affect Ca^2+^-binding affinity or folding stability (Table 1), consistent with a lack of myristate contact by these residues in recoverin.

### Ca^2+^-induced Extrusion of the Myristoyl Group

**Fig 7:**
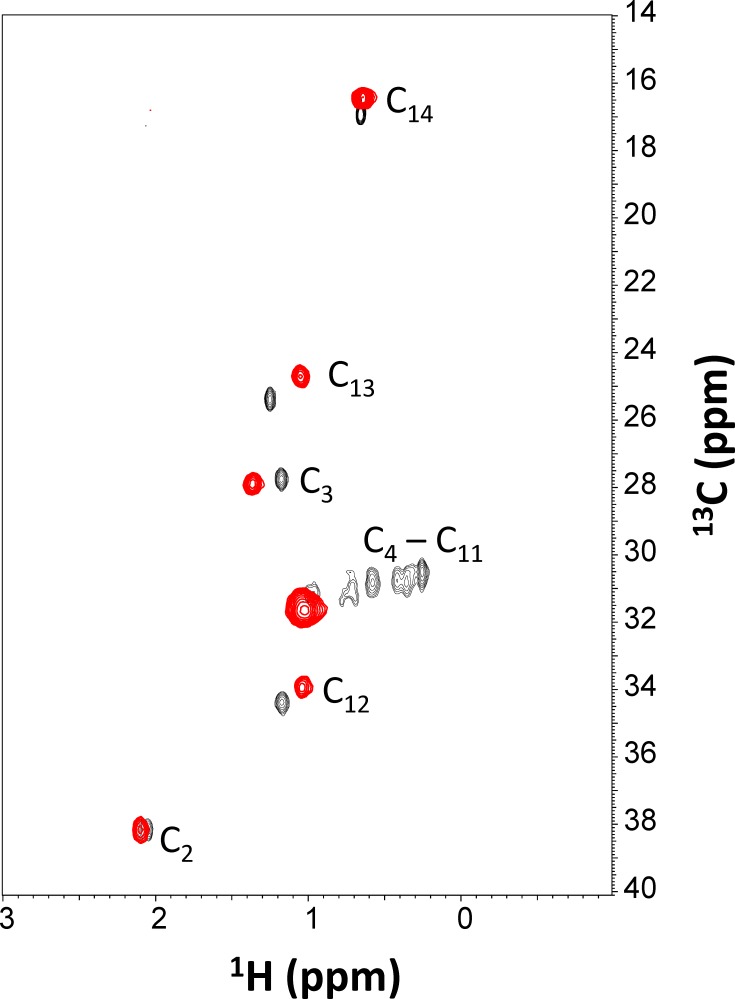
Ca^2+^-induced extrusion of myristate. Overlay of two dimensional ^1^H-^13^C HMQC NMR spectra of the ^13^C-labeled myristoyl group attached to unlabeled Ca^2+^-free VILIP-3 (black peaks) and Ca^2+^ -bound VILIP-3 (red peaks). The spectral changes reflect Ca^2+^-induced environmental changes around the myristoyl group, indicative of Ca^2+^-induced extrusion of the myristate. Chemical shift assignments are provided in Table 3.

To probe Ca^2+^-induced structural changes to the attached myristoyl group, two-dimensional ^1^H-^13^C HMQC experiments were performed on a VILIP-3 sample that contained a ^13^C-labeled myristoyl group attached to unlabeled VILIP-3 (Fig 7). The ^1^H-^13^C HMQC experiment detects protons of myristate that are covalently attached to ^13^C and therefore only NMR resonances of the myristoyl group appear in the spectra. The HMQC spectrum of the ^13^C-labeled myristoyl group attached to Ca^2+^-free VILIP-3 exhibited the expected number of well resolved resonances (see chemical shift assignments in Table 3). The myristate resonances at positions 2, 3, 12, 13 and 14 were unambiguously assigned based on characteristic ^13^C chemical shifts and these resonances formed dipolar interactions with nearby protein residues in Ca^2+^-free VILIP-3 (Fig 6). The upfield shifted proton chemical shifts observed for H12, H13 and H14 of the myristate are consistent with the close proximity of these atoms to aromatic side chains (F48, Y52, F55, F85) inside the VILIP-3 hydrophobic core. The myristate NMR data are therefore consistent with the sequestration of the attached myristoyl group inside Ca^2+^-free VILIP-3 (Fig 6). The ^1^H-^13^C HMQC spectrum of Ca^2+^-bound VILIP-3 reveals significant chemical shift changes to the myristate resonances (Fig 7 and Table 3). The methylene resonances at positions C_4_-C_11_ all collapse into a single peak, suggesting that the covalently attached myristate becomes located in a more solvent exposed environment in the Ca^2+^-bound protein. The chemical shifts of the methylene resonances from the myristoyl group attached to Ca^2+^-bound VILIP-3 are all quite similar to those of free myristic acid in solution [34]. The NMR data demonstrate that the myristoyl group attached to Ca^2+^-bound VILIP-3 is most likely solvent exposed.

**Table 3:** ^1^H and ^13^C (in parentheses) chemical shift assignments of myristoyl resonances.

Position	Ca^2+^-free VILIP-3^a^ (ppm)	Ca^2+^-bound VILIP-3^b^ (ppm)
C2	2.12, 2.06 (38.0)	2.1 (38.2)
C3	1.17 (27.7)	1.4 (28.1)
C4 –C11	0.26–1.00 (30.5–31.2)	1.0 (31.5)
C12	0.98 (34.0)	1.2 (34.0)
C13	1.26 (25.3)	1.1 (24.5)
C14	0.66 (16.9)	0.65 (16.5)

The NMR sample conditions were 0.2 mM VILIP-3 in 10 mM Tris at pH 7.4 containing either 5 mM EDTA ^a^ or 5 mM Ca^2+ b^.

## Discussion

In this study, we determined the energetics of Ca^2+^ binding (Fig 2) and folding (Fig 3) of VILIP-3 as well as the NMR structure of Ca^2+^-free VILIP-3 (Fig 5). Ca^2+^ binds cooperatively (Hill slope of 1.8) to myristoylated VILIP-3 in the sub-micromolar range (ΔH = -6.4 kcal/mol and K_D_ = 0.52 μM). A Hill coefficient of 1.8 is consistent with 3 Ca^2+^ binding sites in VILIP-3 having positive cooperativity, which resembles the cooperative Ca^2+^ binding observed for myristoylated recoverin [33]. Ca^2+^-free myristoylated VILIP-3 has a higher unfolding temperature than the Ca^2+^-free unmyristoylated protein, consistent with protein stabilization caused by the covalently attached myristoyl group. The fatty acyl group is sequestered inside a unique hydrophobic core of Ca^2+^-free VILIP-3 that involves myristate contacts to E26, Y64 and V68 (Fig 5) that are not seen in recoverin [15], NCS-1 [13] and GCAP-1 [12]. The sequestered myristoyl group in VILIP-3 forms an unusual L-shaped conformation with a 90° bend at C_7_ (Fig 6E) and the bent fatty acyl chain makes contact with a shallow protein cavity lined by residues solely in the N-terminal domain (EF1 and EF2). By contrast, the myristoyl group in recoverin is buried in a deeper protein cavity and makes more extensive contact with the protein. The shallower myristate binding pocket in VILIP-3 and fewer protein-myristate contacts may explain its 30-fold higher Ca^2+^-binding affinity and lower folding stability compared to myristoylated recoverin (Table 1).

The distinctive structure of Ca^2+^-free VILIP-3 (Fig 5) is consistent with the idea that N-terminal myristoylation helps to forge each NCS protein into a unique three-dimensional fold [23]. The different structures of the Ca^2+^-free forms of recoverin [15], NCS-1 [13], GCAP1 [11] and VILIP-3 (this study) imply that the Ca^2+^-free states of NCS proteins may have diverse functional activity. Indeed, the Ca^2+^-free state of GCAP1 binds and activates retinal guanylyl cyclases [37,38]. Ca^2+^-free DREAM binds to specific DNA sequences [39–43] and blocks transcription [44]. And Ca^2+^-free calmodulin binds to IQ motifs in numerous target proteins [45–47]. Accordingly, we suggest that the Ca^2+^-free state of VILIP-3 and the other NCS proteins may also bind to specific target proteins and possess distinct biological functions. Future studies are needed to look for target proteins that bind to Ca^2+^-free VILIP-3 and the other NCS proteins. We propose that N-terminal myristoylation plays an important role in creating unique Ca^2+^-free structures of NCS proteins that could provide a means of generating functional diversity.

The physiological target proteins that bind to VILIP-3 are currently not known. Hippocalcin, a close homolog of VILIP-3 (94% identity), binds and regulates Ca^2+^-gated sAHP channels in hippocampal neurons that are important for learning and memory [10]. The very high sequence identity between hippocalcin and VILIP-3 suggests that VILIP-3 could also bind to sAHP channels. Similar to hippocalcin, VILIP-3 may also serve as a Ca^2+^ sensor important for regulating long-term depression (LTD) and hippocampal synaptic plasticity in learning and memory. The Ca^2+^-dependent regulation of sAHP channels mediated by hippocalcin, therefore, might be similar to Ca^2+^-dependent regulation of voltage-gated Ca^2+^ channels mediated by CaM [48,49]. The Ca^2+^-free and Ca^2+^-bound forms of CaM each bind to separate sites on the CaV1.3 channel [49]. Apo-CaM binds to the C-terminal regulatory region of CaV1.3, which promotes channel activation [48]. By contrast, Ca^2+^-bound CaM binds to an N-terminal site (called NSCaTE), which is responsible for inhibiting channel activity [50,51]. Hippocalcin and VILIP-3 might bind and regulate sAHP channels in a similar fashion. Future studies are needed to test whether VILIP-3 binds directly to sAHP channels and find out whether the Ca^2+^-free and Ca^2+^-bound forms of VILIP-3 both bind to distinct regulatory sites on channel targets.

## References

[1] Braunewell KH, Gundelfinger ED (1999) Intracellular neuronal calcium sensor proteins: a family of EF-hand calcium-binding proteins in search of a function. Cell Tissue Res 295: 1–12. 9931348 10.1007/s004410051207

[2] Burgoyne RD (2007) Neuronal calcium sensor proteins: generating diversity in neuronal Ca2+ signalling. Nat Rev Neurosci 8: 182–193. 10.1038/nrn2093 17311005 PMC1887812

[3] Burgoyne RD, O'Callaghan DW, Hasdemir B, Haynes LP, Tepikin AV (2004) Neuronal Ca2+-sensor proteins: multitalented regulators of neuronal function. Trends Neurosci 27: 203–209. 10.1016/j.tins.2004.01.010 15046879

[4] Burgoyne RD, Weiss JL (2001) The neuronal calcium sensor family of Ca2+-binding proteins. Biochem J 353: 1–12. 11115393 PMC1221537

[5] Bernstein HG, Baumann B, Danos P, Diekmann S, Bogerts B, Gundelfinger ED (1999) Cellular Distribution of neural visinin-like protein immunoreactivities in human brain. J Neurocytol 28: 655–662. 10851344 10.1023/a:1007056731551

[6] Spilker C, Gundelfinger E, Braunewell K (2002) Evidence for different functional properties of the neuronal calcium sensor proteins VILIP-1 and VILIP-3: from subcellular localization to cellular function. Biochim Biophys Acta 1600: 118–127. 12445467 10.1016/s1570-9639(02)00452-1

[7] Paterlini M, Revilla V, Grant AL, Wisden W (2000) Expression of the neuronal calcium sensor protein family in the rat brain. Neuroscience 99: 205–216. 10938426 10.1016/s0306-4522(00)00201-3

[8] Spilker C, Richter K, Smalla K, Manahan-Vaughan D, Gundelfinger E, Braunewell K (2000) The neuronal EF-hand calcium-binding protein visinin-like protein-3 is expressed in cerebellar Purkinje cells and shows a calcium-dependent membrane association. Neuroscience 96: 121–129. 10683417 10.1016/s0306-4522(99)00536-9

[9] Kim KS, Kobayashi M, Takamatsu K, Tzingounis AV (2012) Hippocalcin and KCNQ channels contribute to the kinetics of the slow afterhyperpolarization. Biophys J 103: 2446–2454. 10.1016/j.bpj.2012.11.002 23260046 PMC3525844

[10] Tzingounis AV, Kobayashi M, Takamatsu K, Nicoll RA (2007) Hippocalcin gates the calcium activation of the slow afterhyperpolarization in hippocampal pyramidal cells. Neuron 53: 487–493. 10.1016/j.neuron.2007.01.011 17296551 PMC1832111

[11] Lim S, Peshenko IV, Olshevskaya EV, Dizhoor AM, Ames JB (2016) Structure of Guanylyl Cyclase Activator Protein 1 (GCAP1) Mutant V77E in a Ca2+-free/Mg2+-bound Activator State. J Biol Chem 291: 4429–4441. 10.1074/jbc.M115.696161 26703466 PMC4813471

[12] Stephen R, Bereta G, Golczak M, Palczewski K, Sousa MC (2007) Stabilizing function for myristoyl group revealed by the crystal structure of a neuronal calcium sensor, guanylate cyclase-activating protein 1. Structure 15: 1392–1402. 10.1016/j.str.2007.09.013 17997965 PMC2556213

[13] Lim S, Strahl T, Thorner J, Ames JB (2011) Structure of a Ca2+-myristoyl switch protein that controls activation of a phosphatidylinositol 4-kinase in fission yeast. J Biol Chem 286: 12565–12577. 10.1074/jbc.M110.208868 21288895 PMC3069458

[14] Ames JB, Ishima R, Tanaka T, Gordon JI, Stryer L, Ikura M (1997) Molecular mechanics of calcium-myristoyl switches. Nature 389: 198–202. 10.1038/38310 9296500

[15] Tanaka T, Ames JB, Harvey TS, Stryer L, Ikura M (1995) Sequestration of the membrane-targeting myristoyl group of recoverin in the calcium-free state. Nature 376: 444–447. 10.1038/376444a0 7630423

[16] Chen KC, Wang LK, Chang LS (2009) Regulatory elements and functional implication for the formation of dimeric visinin-like protein-1. J Pept Sci 15: 89–94. 10.1002/psc.1097 19065602

[17] Dizhoor AM, Chen CK, Olshevskaya E, Sinelnikova VV, Phillipov P, Hurley JB (1993) Role of the acylated amino terminus of recoverin in Ca(2+)-dependent membrane interaction. Science 259: 829–832. 8430337 10.1126/science.8430337

[18] Kobayashi M, Takamatsu K, Saitoh S, Noguchi T (1993) Myristoylation of hippocalcin is linked to its calcium-dependent membrane association properties. Journal of Biological Chemistry 268: 18898–18904. 8360179

[19] Ladant D (1995) Calcium and membrane binding properties of bovine neurocalcin expressed in *Escherichia coli*. J Biol Chem 270: 3179–3185. 7852401

[20] Spilker C, Dresbach T, Braunewell KH (2002) Reversible translocation and activity-dependent localization of the calcium-myristoyl switch protein VILIP-1 to different membrane compartments in living hippocampal neurons. J Neurosci 22: 7331–7339. 12196554 10.1523/JNEUROSCI.22-17-07331.2002 PMC6757958

[21] Zozulya S, Stryer L (1992) Calcium-myristoyl protein switch. Proc Natl Acad Sci USA 89: 11569–11573. 1454850 10.1073/pnas.89.23.11569 PMC50594

[22] Spilker C, Braunewell K-H (2003) Calcium–myristoyl switch, subcellular localization, and calcium-dependent translocation of the neuronal calcium sensor protein VILIP-3, and comparison with VILIP-1 in hippocampal neurons☆. Molecular and Cellular Neuroscience 24: 766–778. 14664824 10.1016/s1044-7431(03)00242-2

[23] Ames JB, Lim S (2012) Molecular structure and target recognition of neuronal calcium sensor proteins. Biochim Biophys Acta 1820: 1205–1213. 10.1016/j.bbagen.2011.10.003 22020049 PMC3266469

[24] Li C, Pan W, Braunewell KH, Ames JB (2011) Structural Analysis of Mg2+ and Ca2+ Binding, Myristoylation, and Dimerization of the Neuronal Calcium Sensor and Visinin-like Protein 1 (VILIP-1). J Biol Chem 286: 6354–6366. 10.1074/jbc.M110.173724 21169352 PMC3057812

[25] Wingard JN, Chan J, Bosanac I, Haeseleer F, Palczewski K, Ikura M, et al(2005) Structural analysis of Mg2+ and Ca2+ binding to CaBP1, a neuron-specific regulator of calcium channels. J Biol Chem 280: 37461–37470. 10.1074/jbc.M508541200 16147998 PMC1470661

[26] Clore GM, Gronenborn AM (1997) NMR structures of proteins and protein complexes beyond 20,000 M(r). Nat Struct Biol 4: 849–853. 9377157

[27] Delaglio F, Grzesiek S, Vuister GW, Zhu G, Pfeiffer J, Bax A (1995) NMRPipe: a multidimensional spectral processing system based on UNIX pipes. J Biomol NMR 6: 277–293. 8520220 10.1007/BF00197809

[28] Shen Y, Delaglio F, Cornilescu G, Bax A (2009) TALOS+: a hybrid method for predicting protein backbone torsion angles from NMR chemical shifts. J Biomol NMR 44: 213–223. 10.1007/s10858-009-9333-z 19548092 PMC2726990

[29] Schwieters CD, Kuszewski JJ, Tjandra N, Clore GM (2003) The Xplor-NIH NMR molecular structure determination package. J Magn Reson 160: 65–73. 12565051 10.1016/s1090-7807(02)00014-9

[30] Badger J, Kumar RA, Yip P, Szalma S (1999) New features and enhancements in the X-PLOR computer program. Proteins 35: 25–33. 10090283

[31] Nilges M, Gronenborn AM, Brunger AT, Clore GM (1988) Determination of three-dimensional structures of proteins by simulated annealing with interproton distance restraints. Application to crambin, potato carboxypeptidase inhibitor and barley serine proteinase inhibitor 2. Protein Eng 2: 27–38. 2855369 10.1093/protein/2.1.27

[32] Bagby S, Harvey TS, Eagle SG, Inouye S, Ikura M (1994) NMR-derived three-dimensional solution structure of protein S complexed with calcium. Structure 2: 107–122. 8081742 10.1016/s0969-2126(00)00013-7

[33] Ames JB, Porumb T, Tanaka T, Ikura M, Stryer L (1995) Amino-terminal myristoylation induces cooperative calcium binding to recoverin. J Biol Chem 270: 4526–4533. 7876221 10.1074/jbc.270.9.4526

[34] Ames JB, Tanaka T, Ikura M, Stryer L (1995) Nuclear magnetic resonance evidence for Ca(2+)-induced extrusion of the myristoyl group of recoverin. J Biol Chem 270: 30909–30913. 8537345 10.1074/jbc.270.52.30909

[35] Li C, Ames JB (2014) (1)H, (1)(3)C, and (1)(5)N chemical shift assignments of neuronal calcium sensor protein, hippocalcin. Biomol NMR Assign 8: 63–66. 10.1007/s12104-012-9453-3 23250791 PMC3625700

[36] Zhang M, Tanaka T, Ikura M (1995) Calcium-induced conformational transition revealed by the solution structures of apo calmodulin. Nat Struct Biol 2: 758–767. 7552747 10.1038/nsb0995-758

[37] Dizhoor AM, Lowe DG, Olsevskaya EV, Laura RP, Hurley JB (1994) The human photoreceptor membrane guanylyl cyclase, RetGC, is present in outer segments and is regulated by calcium and a soluble activator. Neuron 12: 1345–1352. 7912093 10.1016/0896-6273(94)90449-9

[38] Palczewski K, Subbaraya I, Gorczyca WA, Helekar BS, Ruiz CC, Ohguro H, et al (1994) Molecular cloning and characterization of retinal photoreceptor guanylyl cyclase-activating protein. Neuron 13: 395–404. 7520254 10.1016/0896-6273(94)90355-7

[39] Gomez-Villafuertes R, Torres B, Barrio J, Savignac M, Gabellini N, Rizzato F, et al (2005) Downstream regulatory element antagonist modulator regulates Ca2+ homeostasis and viability in cerebellar neurons. J Neurosci 25: 10822–10830. 10.1523/JNEUROSCI.3912-05.2005 16306395 PMC6725879

[40] Osawa M, Dace A, Tong KI, Valiveti A, Ikura M, Ames JB(2005) Mg2+ and Ca2+ differentially regulate DNA binding and dimerization of DREAM. J Biol Chem 280: 18008–18014. 10.1074/jbc.M500338200 15746104

[41] Osawa M, Tong KI, Lilliehook C, Wasco W, Buxbaum JD, Cheng HY, et al(2001) Calcium-regulated DNA binding and oligomerization of the neuronal calcium-sensing protein, calsenilin/DREAM/KChIP3. J Biol Chem 44: 41005–41013. 10.1074/jbc.M105842200 11535596

[42] Scsucova S, Palacios D, Savignac M, Mellstrom B, Naranjo JR, Ananda A(2005) The repressor DREAM acts as a transcriptional activator on Vitamin D and retinoic acid response elements. Nucleic Acids Res 33: 2269–2279. 10.1093/nar/gki503 15849313 PMC1084319

[43] Tiruppathi C, Soni D, Wang DM, Xue J, Singh V, Thippegowda PB, et al(2014) The transcription factor DREAM represses the deubiquitinase A20 and mediates inflammation. Nat Immunol 15: 239–247. 10.1038/ni.2823 24487321 PMC4005385

[44] Carrion AM, Link WA, Ledo F, Mellstrom B, Naranjo JR (1999) DREAM is a Ca2+-regulated transcriptional repressor. Nature 398: 80–84. 10.1038/18044 10078534

[45] Chagot B, Chazin WJ (2011) Solution NMR structure of Apo-calmodulin in complex with the IQ motif of human cardiac sodium channel NaV1.5. J Mol Biol 406: 106–119. 10.1016/j.jmb.2010.11.046 21167176 PMC3030672

[46] Feldkamp MD, Yu L, Shea MA (2011) Structural and energetic determinants of apo calmodulin binding to the IQ motif of the Na(V)1.2 voltage-dependent sodium channel. Structure 19: 733–747. 10.1016/j.str.2011.02.009 21439835 PMC3094505

[47] Hoffman L, Chandrasekar A, Wang X, Putkey JA, Waxham MN (2014) Neurogranin alters the structure and calcium binding properties of calmodulin. J Biol Chem 289: 14644–14655. 10.1074/jbc.M114.560656 24713697 PMC4031520

[48] Adams PJ, Ben-Johny M, Dick IE, Inoue T, Yue DT (2014) Apocalmodulin itself promotes ion channel opening and Ca(2+) regulation. Cell 159: 608–622. 10.1016/j.cell.2014.09.047 25417111 PMC4349394

[49] Ben Johny M, Yang PS, Bazzazi H, Yue DT (2013) Dynamic switching of calmodulin interactions underlies Ca2+ regulation of CaV1.3 channels. Nat Commun 4: 1717 10.1038/ncomms2727 23591884 PMC3856249

[50] Dick IE, Tadross MR, Liang H, Tay LH, Yang W, Yue DT(2008) A modular switch for spatial Ca2+ selectivity in the calmodulin regulation of CaV channels. Nature 451: 830–834. 10.1038/nature06529 18235447 PMC4262256

[51] Liu Z, Vogel HJ (2012) Structural basis for the regulation of L-type voltage-gated calcium channels: interactions between the N-terminal cytoplasmic domain and Ca(2+)-calmodulin. Front Mol Neurosci 5: 38 10.3389/fnmol.2012.00038 22518098 PMC3324987

